# Personalized behavior change program for glaucoma patients with poor adherence: a pilot interventional cohort study with a pre-post design

**DOI:** 10.1186/s40814-018-0320-6

**Published:** 2018-07-23

**Authors:** Paula Anne Newman-Casey, Leslie M. Niziol, Chamisa K. Mackenzie, Kenneth Resnicow, Paul P. Lee, David C. Musch, Michele Heisler

**Affiliations:** 1 Department of Ophthalmology and Visual Sciences, University of Michigan Medical School, 1000 Wall Street, Ann Arbor, MI 48105 USA; 2 Department of Health Behavior and Health Education, University of Michigan School of Public Health, Ann Arbor, MI 48105 USA; 3 Department of Epidemiology, University of Michigan School of Public Health, Ann Arbor, MI 48105 USA; 4 Department of Internal Medicine, University of Michigan Medical School, Ann Arbor, MI 48109 USA

**Keywords:** Glaucoma, Education, Counseling, Adherence, Motivational interviewing, Tailoring

## Abstract

**Background:**

About half of people with glaucoma do not adhere to their recommended medications. Interventions for other chronic conditions have successfully utilized reminder systems and motivational interviewing (MI)-based counseling. This study was designed to pilot a personalized intervention that leverages these strategies to assess their impact on medication adherence in glaucoma patients.

**Methods:**

Glaucoma patients taking ≥ 1 medication will be pre-screened by telephone survey for adherence to their medication(s). Those who self-report poor adherence will be enrolled in a 3-month monitoring period to measure medication adherence using electronic medication monitors. Participants who are non-adherent (take </=80% of their medication doses) over the 3-month run in phase will be eligible for the study. We plan to enroll 57 participants who are non-adherent to their medications. Participants’ adherence will then be continuously measured with electronic medication monitors, by self-report, and via pharmacy refill data over 2 years, during which two successively more resource-intensive components of an intervention aimed to improve medication adherence will be administered. The first component is a 3-month period of reminders (audio and/or visual) and text message or automated phone call if a dose of medication is not taken within a pre-specified time frame. The second component is a 6-month MI-based counseling program with a trained glaucoma counselor. This component uses the *eyeGuide*, a computer-based personalized behavior change program that enables para-professional staff to provide personalized education and counseling for glaucoma. The primary outcome is change in medication adherence. The secondary outcomes include changes in clinical outcomes (intraocular pressure, IOP, and IOP fluctuation) and psychosocial mediators of adherence (e.g., competence, energy for change and satisfaction). Participants will undergo semi-structured interviews at 12 months to give feedback about the counseling program in order to improve it.

**Discussion:**

This pilot study will provide insight into ways to deliver more personalized health care to non-adherent glaucoma patients in order to better support them in managing their chronic disease.

**Trial registration:**

Retrospectively registered with ClinicalTrials.gov (NCT03159247).

**Electronic supplementary material:**

The online version of this article (10.1186/s40814-018-0320-6) contains supplementary material, which is available to authorized users.

## Background

Despite evidence from randomized clinical trials that medication reduces vision loss from glaucoma [1, 2], it remains the second leading cause of blindness in the United States (US) [3]. One major contributor to this is that about one-half of glaucoma patients are essentially “untreated” because they do not adhere to their medications [4, 5]. Poor adherence and poor clinical outcomes disproportionately affect the most vulnerable members of US society, older people, and minorities [6, 7]. As life expectancy in the US continues to increase, the prevalence of glaucoma will also increase. As there is already a projected shortage of ophthalmologists in the workforce [8], we will need to re-think the current paradigm of how a single physician is responsible for medical decision making, surgical intervention, counseling and educating patients, and coordinating care in a complex medical system. Team-based care is becoming essential where a larger team of medical staff help support patients’ chronic disease self-management. There is a compelling need to develop and test technology-based solutions to improving the quality of care a medical team can provide to improve medication adherence and the outcomes of care for patients with glaucoma.

Poor adherence to effective medications is a critical barrier to achieving better outcomes in glaucoma patients. The World Health Organization stated that “increasing the effectiveness of adherence interventions may have a far greater impact on the health of the population than any improvement in specific medical treatments [9].” Once diagnosed with glaucoma, at least half of patients do not adhere to their glaucoma medication regimen [4, 5, 10], return for follow-up [6], or persist with their medications over the longer term [11]. Patients who are not adherent have more severe visual field loss [12–14], which leads to steep declines in health-related quality of life [15–18] and increased risk of falls and motor vehicle accidents [19].

Adherence to glaucoma medications is rarely addressed during the clinical encounter [20, 21] because education and counseling programs are not part of standard glaucoma care. Qualitative research has demonstrated that patients often have a poor understanding of glaucoma and its treatment [22–25]. In addition to knowledge gaps, patients have numerous concrete and psychological barriers to managing their glaucoma such as skepticism that glaucoma will cause vision loss in the future when it is asymptomatic, issues with side effects and medication cost, problems remembering to take medication, and difficulties properly instilling the medication [26–28]. Eye drop instillation is rarely taught during the clinic visit, and many patients cannot properly instill their drops [29, 13]. For example, among glaucoma patients with visual impairment, one-third of patients who thought they could correctly instill their eye drops did not get their eye drop into their eye when they were video-recorded [29].

**Fig. 1:**
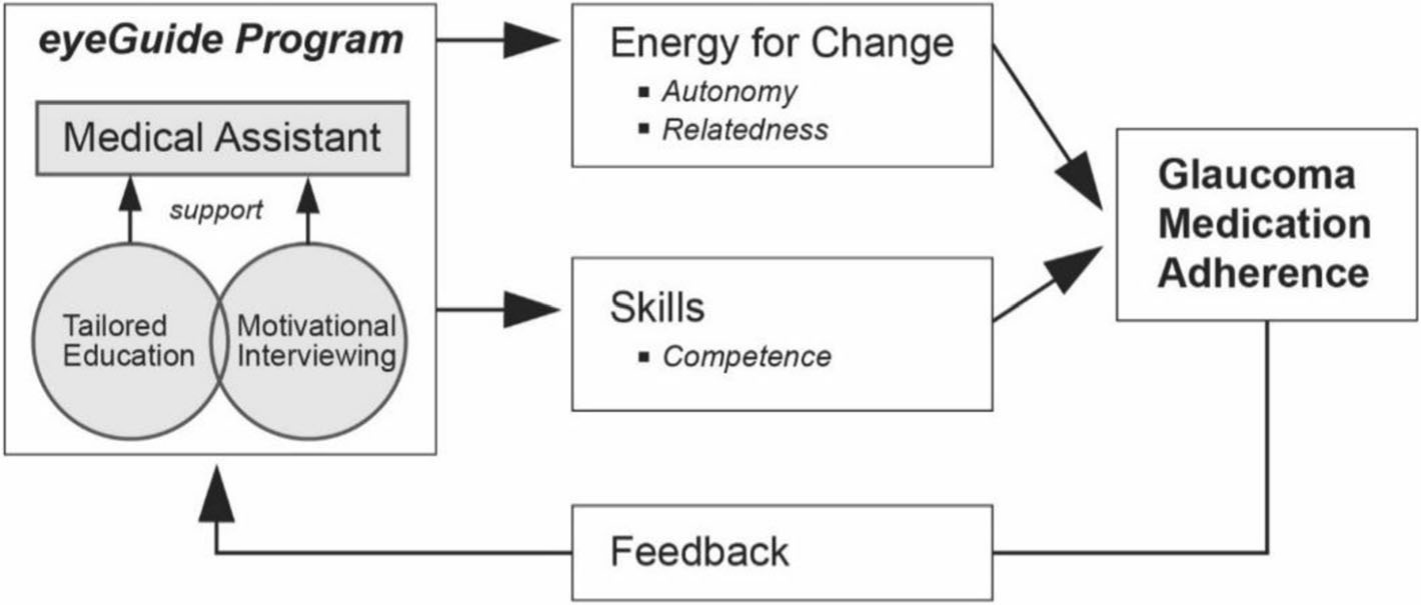
Self-determination theory based theoretical model

Uniform, scripted approaches to improve adherence have been shown to be ineffective [30, 31]. However, complex, individualized counseling interventions, especially those based in motivational interviewing (MI), have been shown to improve adherence and health outcomes in many chronic diseases [30, 31]. MI is a style of counseling that engages patients by discussing priorities and obstacles to facilitate intrinsic motivation to change health behavior [32]. MI is a counseling style consistent with the theoretical framework of self-determination theory [33], which postulates that an individual must develop personally compelling reasons to motivate a change in behavior. Self-motivation depends on meeting three basic psychological needs: relatedness, autonomy, and competence. A collaborative discussion based in MI techniques allows these needs be met and creates “energy for change.” (Fig. 1). Relatedness is promoted by expressing empathy during counseling. Autonomy is supported when the counselor helps the person overcome ambivalence and identify his own values and goals for managing disease. MI supports competence and self-efficacy by equipping the person with the knowledge and skills to work through barriers and implement routines to maintain the behavior change (create an “action plan”). MI and SDT will serve as the health communications framework for the tailored strategies for our interventions in the non-adherent glaucoma patient population. Few complex interventions based on these successful principles have been rigorously tested and none have been implemented into glaucoma care.

**Fig. 2:**
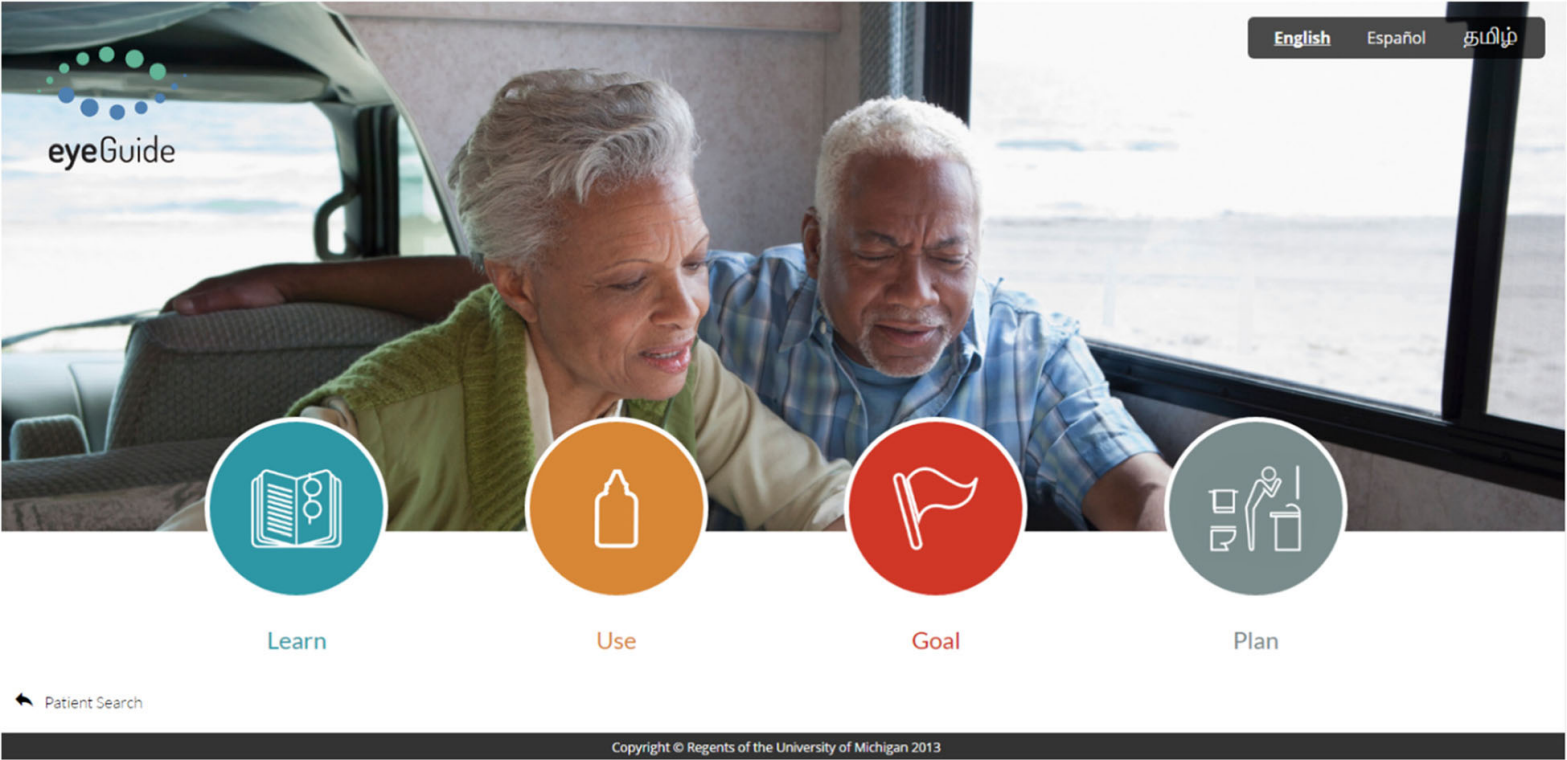
eyeGuide tool screenshots

The eyeGuide is a web-based, personally tailored, behavior change program based on MI principles and self-determination theory, developed from a systematic review of the glaucoma adherence literature [34], data from focus groups [35] and surveys [26], and iterative beta-testing with glaucoma patients [36] (Fig. 2). The eyeGuide was developed with user centered design; we tested the eyeGuide with patients and providers and when more than one person suggested something should be changed, we changed it. We continued this process until we no longer received substantive comments from patients and providers, which occurred after testing with 40 patients and 8 providers through three major design changes. The eyeGuide has two components woven together into a single web-based tool: an electronic health (eHealth) component and a semi-structured, tailored interview guide to facilitate an MI-based conversation. The eHealth component provides individually tailored disease and treatment information, and information on how other patients overcame similar barriers to optimize their disease self-management. Such technology-based eHealth innovations have great potential to extend the reach of physicians by enabling team-based care.

In this pre-post design pilot study, we will test the initial impact of two sequentially more resource-intensive components of a personalized eHealth intervention on medication adherence among non-adherent glaucoma patients: (1) real-time automated adherence reminders and (2) the eyeGuide MI-based counseling program. We will also explore the preliminary effects of these interventions on secondary outcomes including psychosocial mediators of adherence (e.g., competence, energy for change, satisfaction) and clinical parameters (intraocular pressure (IOP) and IOP fluctuation).

## Methods/design

In order to test the preliminary impact of the personalized eHealth intervention among non-adherent glaucoma patients, we will electronically monitor medication adherence longitudinally over 2 years. The two main components to be evaluated are (1) real-time automated adherence reminders using commercially available AdhereTech electronic medication monitors (AdhereTech, New York, USA) and (2) in-person tailored counseling with a medical assistant trained in MI (the eyeGuide program). Adherence will be monitored for all patients for a total of 2 years. Adherence will first be monitored for a 3-month period prior to the intervention to obtain baseline adherence and mitigate issues with regression to the mean and the Hawthorne effect [37]. Adherence will then be measured over a nine-month period of time during which the two interventions will be sequentially administered. Finally, adherence will be measured for 1 year after the conclusion of intervention to assess how well change in adherence is maintained over time.

### Ethics, consent, and permissions

Approval for the study was obtained from the University of Michigan Institutional Review Board (HUM00112614). Eligible participants will be assessed for willingness to participate in the study. Informed consent will be obtained at the first study visit for study participation alongside permission to access health information from the medical record and pharmacy refill data from participants’ pharmacies.

### Participants

Participants who have been seen at the University of Michigan Kellogg Eye Center, have a diagnosis of glaucoma (including glaucoma suspect and ocular hypertension), are aged ≥ 40, take ≥ 1 glaucoma medication, and speak English will be eligible to participate in the study. Eligible glaucoma patients will be sent a letter explaining the study along with an option to opt out of recruitment. Those who do not opt out will be called and their adherence status will be assessed by two survey methods of self-report to increase the probability of recruiting truly non-adherent patients (see Additional file 1 for measures to assess self-reported medication adherence). We will exclude patients who do not administer their own eye drops or who have a diagnosis of cognitive impairment or severe mental illness.

### Baseline eligibility assessment

Adherence will be measured electronically for 3 months prior to the first intervention to determine study eligibility and obtain a baseline, pre-intervention measure of medication adherence. The participant’s medication(s) and dosing schedule is determined by first reviewing the patient’s medical record and then confirming the medications with the patient. The study coordinator will record these data into the AdhereTech system. Participants will place their glaucoma medications inside AdhereTech electronic medication monitors that look like pill bottles. Patients will have different monitors for each of their glaucoma medications. Each time a bottle is opened, and the time and date are recorded. These records will be collected in real time and transmitted through the cellular data network so that adherence to medications can be calculated at any time interval (weekly, biweekly, monthly, etc.). The study coordinator will record each participant’s adherence data in the medical record during their study visits.

An adherent event is defined as taking medication within a specified time window of a dose on the previous day. For a once daily medication, an adherent event is defined as taking the medication within 24 ± 4 h of the previous day’s dose. For a twice daily medication, an adherent event is defined as taking the first medication dose within 24 ± 2 h of the previous day’s first dose and taking the second medication dose within 24 ± 2 h of the previous day’s second dose. For a three times daily medication, an adherent event is defined as taking the medication dose (first, second, or third) within 24 ± 1.3 h of the previous day’s corresponding medication dose (first, second, or third). For a four-time daily medication, an adherent event is defined as taking the medication dose (first, second, third, or fourth) within 24 ± 1 h of the previous day’s corresponding medication dose. The biological efficacy of medications dosed multiple times daily declines when not taken on time [38–40]. However, when calculating adherence for medications dosed more than once a day, we compare the current day’s doses to the previous day’s corresponding doses rather than simply the previous dose (second versus first, or third versus second) as lifestyle and sleeping patterns can result in medication times that are not equally spaced. This method of calculating adherence also allows for large shifts (time zone changes for work or vacation) or gradual changes in times when medications are taken without overly penalizing the patient, which can happen when calculating adherence relative to a median time. Additionally, this method of measuring adherence ensures that adherence credit is not given for instances where bottles are opened numerous times just prior to a clinic visit [119]. For participants on more than one medication, adherence will first be measured at the medication level and then aggregated to the person level by dividing the total number of doses of all medication(s) taken on time by the total number of doses of all medication(s) prescribed. Baseline adherence will be calculated monthly during the 3-month monitoring period. The median monthly adherence will be designated as the baseline adherence score; choosing the median will help to mitigate the effects of regression to the mean. We will exclude patients from participating in the study whose median baseline adherence score is > 80% on all of their glaucoma medications after a baseline observation period of 3 months.

### Timeline

**Fig. 3:**
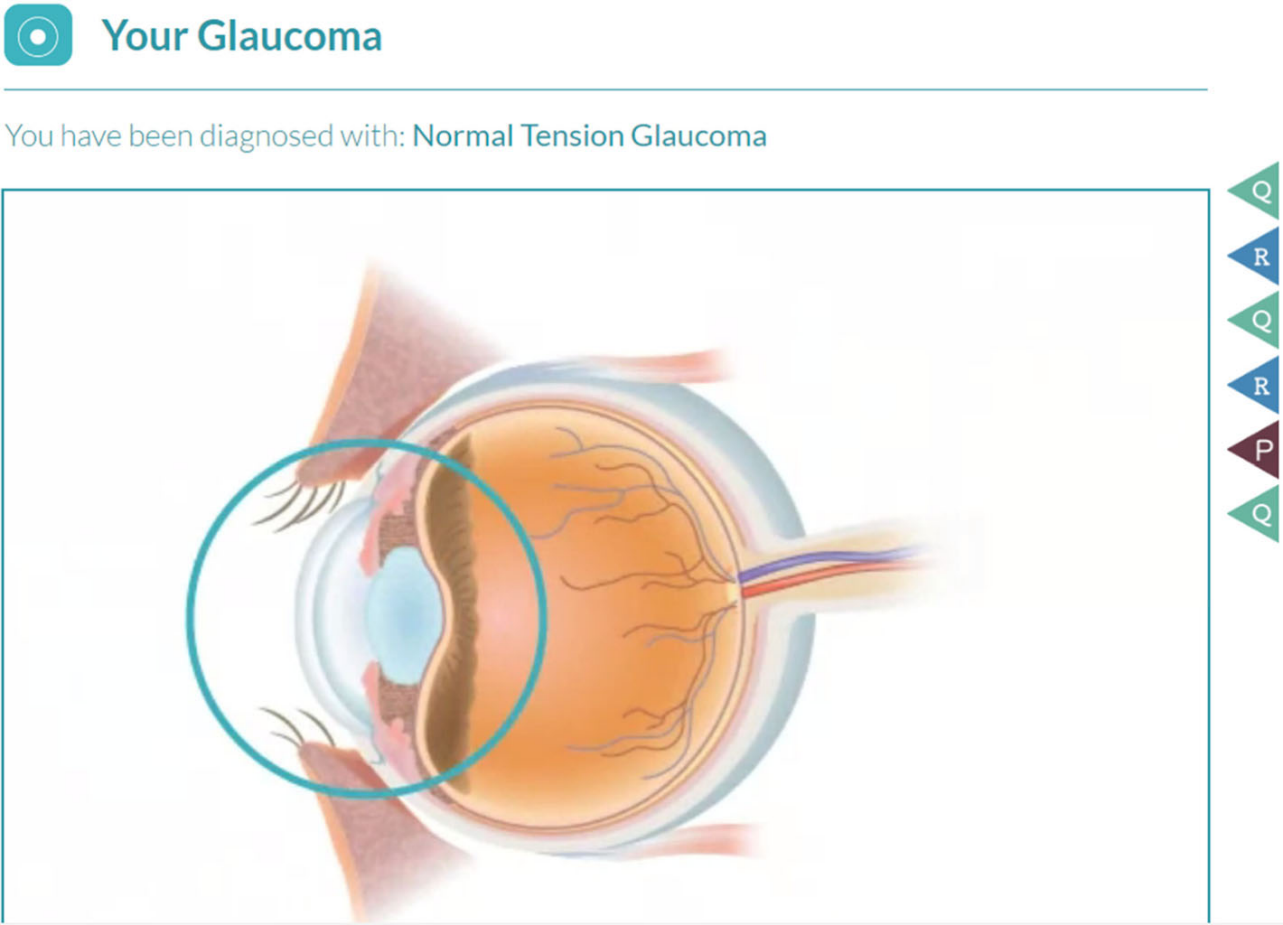
Pilot study timeline

The glaucoma counselor will meet with the participants in-person for seven study visits (Fig. 3). Intraocular pressure (IOP) will be measured at each study visit. The first study visit will include obtaining written informed consent, taking a 60-min survey and being instructed on how to use the AdhereTech electronic medication monitors. Three months later, during the second study visit, participants will be notified of their eligibility to continue in the study.

Eligible patients will participate in five more study visits. At the third study visit, 6 months from enrollment, participants will have their first 60-min eyeGuide counseling session with the glaucoma counselor and will take a 30-min survey. Their eye drop instillation will also be video-recorded. At the fourth study visit, 8 months from enrollment, participants will have their second 30-min eyeGuide counseling session and will take a 10-min survey. At their fifth study visit, 10 months from enrollment, they will have their final 30 min eyeGuide counseling session and have their eye drop instillation technique video-recorded. The intervention will end at the sixth study visit, 12 months from enrollment, where participants will come in to take a 45-min survey and undergo a 30-min semi-structured interview to obtain qualitative feedback about the eyeGuide program. Between the in-person eyeGuide counseling sessions, the glaucoma counselor will check-in with participants by telephone to update them on their adherence and discuss any issues they are having. These check-ins will take place more frequently at the beginning of the eyeGuide intervention, at 2 week intervals, and less frequently toward the end of the eyeGuide intervention (at 1 month intervals; Fig. 3). Participants will continue using their electronic medication monitors for an additional 12 months after the intervention has ended. At the seventh and final study visit, 24 months from enrollment, participants will return their electronic medication monitors and take a 45-min survey. Participants will receive a $35 stipend for each of their seven study visits and $35 for returning their adherence monitors.

### Personalized intervention tested in a pre-post design

#### Component 1

Participants will decide on a daily time when they would like to receive an automated reminder to take each dose of all of their glaucoma medications *if* they have not yet taken the dose. The reminder will be either an automated phone call or text message. Participants can also choose to activate an audio or visual alert [41–43]. AdhereTech will provide all of the preferred automated alerts. Participants’ adherence will be monitored throughout the 3-month period of receiving these automated reminders.

#### Component 2

After 3 months of receiving the automated reminders, participants will begin the tailored eyeGuide program. The program is tailored using computer programming so that the information each participant receives is personalized to their demographics and circumstances. The eyeGuide is tailored on the following variables: name, gender, race/ethnicity, ophthalmologist’s name, ophthalmologist’s gender, glaucoma type, past glaucoma surgeries or lasers, optic nerve photographs, visual field test results, use of the internet and/or smartphone, family support for eye drop instillation, and barriers to optimal glaucoma medication adherence.

Using the eyeGuide tablet-based tool, the glaucoma counselor will teach eye drop instillation, provide education tailored to a participant’s test results, elicit the barriers the participants face in managing their glaucoma, share tailored patient testimonials about strategies other patients used to overcome similar barriers, and brainstorm solutions together during the three in-person counseling sessions. The counselor will collaborate with participants to form an “action plan”—a short-term plan of steps to use to address identified barriers including how to integrate glaucoma medications into daily life (Fig. 2) [44–46]. The counselor will call participants once every 2 weeks between the first two eyeGuide study visits and then once monthly to provide feedback on their adherence level, check-in on their action plan, and help the participant identify new action steps. In-person counseling sessions will take place in a small Wi-Fi-enabled conference room at the Kellogg Eye Center located nearby an examination room where IOP measurement is taken.

Participants will receive 170 min of counseling (one 60-min counseling session, two 30-min counseling sessions, and five 10-min telephone calls), within the range of counseling time (106 ± 92.4 min) that achieved significant results in a meta-analysis of motivational interviewing [46]. The glaucoma counselor will also update the participant’s ophthalmologist on their adherence and action plan. Participants can call the counselor if questions arise during the intervention.

### Glaucoma-specific brief MI training program development

An ophthalmic technician will be trained as a glaucoma counselor through a glaucoma-specific brief MI training program that the study team has developed. The glaucoma-specific brief MI training program was developed by two behavioral psychologists and a psychotherapist trained through the Motivational Interviewing Network of Trainers [47]. The training program includes 16 h of didactic group training and 2 h of individualized coaching sessions. During the first training session, the prospective counselor learns about the following components of brief-MI-based conversation: reflective listening, asking open-ended questions, using affirmations, using elicit-provide-elicit technique to teach eye drop instillation, and summarizing [32]. The elicit-provide-elicit technique includes assessing the patient’s knowledge prior to giving information, asking permission to give new information, and then following up by eliciting patients’ response to the new information to evaluate their understanding.

The second training session focuses on learning how to problem-solve issues that may arise in patient encounters and learning how to make complex reflections. A reflection is a statement that paraphrases what the patient said to demonstrate active listening; a complex reflection is a statement that paraphrases and adds significant meaning. Prospective counselors also learn how to identify and promote change talk and develop an action plan to improve medication adherence. A video collection of glaucoma patients explaining their reasons for discontinuing their glaucoma medications is used for role playing during the training. Each session includes both didactic presentations and role playing (Table 1).

**Table 1:** Glaucoma-specific brief motivational interviewing training curriculum

	Training session 1	Training session 2
Knowledge review	Presentation using “Elicit-Provide-Elicit”: “Motivational Interviewing Basics”	Review using E-P-E: “Skills learned in session 1”
		Skill review: “Quiz open vs. closed questions”
Skills/knowledge development	Paired activity: “A Taste of MI”	Group activity: “Health and Safety Quiz”
	Didactic using E-P-E: “What is reflective listening”	Triad activity: “Ambivalence”
	Paired activity: “Non verbal listening practice”	Didactic using E-P-E: “MI Spirit”
	Paired activity: “Hypothesis testing”	Didactic using E-P-E: “Simple vs. complex reflections”
	Paired activity: “Forming reflections”	Individual activity: “Reflective responses to sentence stems—three levels”
	Individual activity: “Forming reflections with sentence stems”	Paired activity: “Reflection practice”
	Group activity: “Forming reflections to glaucoma patient video examples”	Didactic using E-P-E: “Affirmations”
	Didactic using E-P-E: “Open ended questions”	Paired activity: “Making affirmations”
	Paired activity: “Asking open-ended questions”	Didactic using E-P-E: “Making summaries”
	Didactic using E-P-E: “Elicit-provide-elicit”	Paired activity: “Making summaries”
	Paired activity: “Teaching eye drop instillation with elicit-provide-elicit”	Didactic using E-P-E: “Eliciting change talk”
	Didactic using E-P-E: “Summarizing:	Paired activity: “Using elicit-provide-elicit to teach eye drop instillation”
	Paired activity: “Making summaries”	Didactic using E-P-E: “Importance Ruler”
	Paired activity: “Doing a full patient encounter using the MI skills learned”	Didactic using E-P-E: “Exploring goals and values”
		Paired activity” “Exploring goals and values”
		Didactic using E-P-E: “Confidence Ruler”
		Didactic using E-P-E: “Exploring strengths”
		Paired activity: “Rulers and strengths”
		Didactic using E-P-E: “Change Talk vs. Sustain Talk”
		Didactic using E-P-E: “Action Planning”
		Paired activity: “Putting it all together”
Skills Demonstration	Role playing: “Reflective listening”	Video demonstration: “Making Affirmations”
	Role playing: “Patient encounter using MI”	Video demonstration: “Making summaries”
		Video demonstration: “Elicit-Provide-Elicit for Change Talk”
		Video demonstration: “Exploring goals and values”
		Role play: “Rolling with resistance”
Skills self-reflection	Debrief: “What was learned?”	Debrief: “How will you implement this in clinic”

To ensure fidelity to MI training, the glaucoma counselor will practice delivering the intervention with at least five volunteer patients. S/he will be audio-recorded, and feedback will be provided prior to beginning the study. Competence in MI will be met before study visits are conducted. All study visit counseling sessions will be audio- recorded, and a random sample of sessions will be graded with a validated coding system tailored to the study, the One-Pass coding system [48] (Additional file 2**,** Glaucoma-specific one-pass). In addition to the initial training, the counselor will receive feedback on the graded sessions during booster training sessions over the course of the intervention [49]. The protocol for the eyeGuide intervention meets the Template for Intervention Description and Replication (TiDieR) standards [50].

### Outcomes

#### Primary outcome

The primary outcome is change in medication adherence (continuous variable for percentage of medication doses taken on time) measured by electronic medication monitors after each intervention. We will also measure adherence by three additional techniques in order to reflect different aspects of adherence behavior [51] and compare the techniques to one another: (a) video-recorded drop instillation, (b) self-reported adherence [52–55], and (c) pharmacy refill data. To assess how well patients can properly instill their eye drops, we will video-record patients instilling their eye drops during their third and fifth study visits, before and at the end of the eyeGuide intervention, respectively. Two masked assessors will review the videos and assess each participant for the following three techniques: (1) does the participant dispense a single drop, (2) does the drop get into the eye, and (3) does the participant touch the dropper tip to the ocular adnexa or extraocular structures. Self-reported adherence will be assessed with the Morisky questionnaire [56] (eight items, Cronbach *α* = 0.83), the Adherence to Refills and Medications Scale [57] (ARMS, 12 items, *α* = 0.81), and three additional questions about adherence from the glaucoma literature [55, 51]. These adherence questionnaires will be given at the baseline, 3-, 6-, 12-, and 24-month study visits.

Pharmacy refill data will be obtained by calling each participant’s pharmacy every 3 months to identify whether each prescribed medication has been filled. The medication possession ratio (MPR) is the proportion of days during a fixed time period where the patient had an adequate supply of medication to use at the prescribed frequency. MPR will be calculated at the patient level after combining refill data from all medications during a defined time period, as follows:

$$ \frac{\mathrm{MPR}=\kern0.5em \#\mathrm{days}\ \mathrm{correct}\ \mathrm{amount}\ \mathrm{of}\ \mathrm{medication}\ 1+\dots +\#\mathrm{days}\ \mathrm{correct}\ \mathrm{amount}\ \mathrm{of}\ \mathrm{medication}\ 4}{\#\mathrm{elapsed}\ \mathrm{days}\ \mathrm{medication}\ 1+\dots +\#\mathrm{elapsed}\ \mathrm{days}\ \mathrm{medication}\ 4} $$

In this study, MPR will be calculated during fixed, quarterly time intervals of 91–92 days from enrollment, as well as cumulatively over each intervention period. The participants’ pharmacies will be called after a quarter has been completed to obtain refill information (date of fill, medication dispensed, day’s supply, number of mLs dispensed). In the event of overlapping refills, it will be assumed that medications are taken sequentially, not doubly dosed, to avoid an MPR greater than 1.

#### Secondary outcomes

Secondary patient outcomes include changes in clinical outcomes, changes in psychosocial mediators of adherence, and intervention impact on patient satisfaction. Our clinical outcome is IOP and IOP fluctuation [58], which will be measured during each study visit and collected from clinic visits that participants have during the study period (expect 7–9 measurements from study visits and clinic visits). IOP will be checked at each in-person study visit using Goldmann applanation tonometry. Any additional IOP measurements taken during clinic visits during participants’ time in the study will be abstracted from the electronic health record.

The two psychosocial mediators of adherence in our theoretical model (Fig. 1) include (1) change in perceived *glaucoma skills* (perceived competence [59]), glaucoma knowledge [60], glaucoma medication and eye drop instillation self-efficacy [61], goal setting [62], and propensity to ask questions of their physician [63]) and (2) change in *energy for change* (motivational state [64] and autonomy [65]) after each intervention. We will also assess the intervention’s impact on patient satisfaction (satisfaction with information about glaucoma [66] and client satisfaction [67, 68]).

Validated measures will be used to assess these proposed mediators of adherence. Skills will be measured with five constructs: perceived competence, glaucoma knowledge, self-efficacy, goal setting, and confidence asking the physician questions. Perceived competence [59] (four items, Cronbach *α* = 0.80) assesses participants’ perceptions of how able they are to manage their glaucoma on a seven-point Likert scale. Perceived competence will be measured at the baseline, 6-, 12-, and 24-month study visits. Glaucoma Knowledge will be assessed with a 10-item true/false test generated by the National Eye Institute (NEI) [60]. Glaucoma knowledge will be measured at the 3- and 8-month study visits, before and after the eyeGuide intervention, respectively, and at the 24-month study visit. Glaucoma medication and eye drop instillation self-efficacy [61] (medication self-efficacy, 10 items, eye drop instillation self-efficacy, 6 items, *α* = 0.91 for all 16 items) assess participants’ self-confidence in using their eye drop medications as prescribed and instilling them into their eyes correctly using a three-point Likert scale. Glaucoma medication and eye drop instillation self-efficacy will be measured at the baseline, 6-, 12-, and 24-month study visits. Goal setting (three items, *α* = 0.80) measures whether or not health care providers asked participants to set goals to improve how they take care of their chronic disease on a five-point Likert scale. The Goal Setting Scale is a sub-scale of the Patient Assessment of Chronic Illness [62] measure. We could not use the full sub-scale as the final two questions ask if the person has been asked to attend a class to help cope with their chronic condition or asked to fill out a survey about their chronic condition, neither of which are standard aspects of current glaucoma care. Goal setting will be measured at the baseline study visit, and the 12- and 24-month study visits. Confidence asking the physician questions will be measured with three items from the adapted Perceived Efficacy in Patient-Physician Interactions Scale [63] (PEPPI, five items, α = 0.83) using a five-point Likert scale. Confidence asking the physician questions will be assessed at the baseline visit and the 12-month study visit.

Energy for change will be measured with two constructs using two different scales, the Health Care Climate Questionnaire [65] (HCCQ; 12 items, *α* = 0.96) and the Treatment Self-Regulation Questionnaire [64] (TSRQ; 19 items, *α* = 0.81). The HCCQ measures perceived autonomy support from health care providers. The TSRQ measures whether a participant feels motivated to accomplish a health-related goal because of their own internal desires, such as “I personally believe that controlling my glaucoma will improve my health” or whether they take care of their health problem for reasons external to themselves, like “I want my doctor to think I’m a good patient.” Both of these scales have been validated in patients with diabetes and have been adapted to glaucoma, a similar chronic disease that requires consistent self-management for optimal disease control, for this study. Both scales use seven-point Likert scales. The HCCQ and TSRQ will both be given at the baseline, 6-, 12-, and 24-month study visits.

Satisfaction will be measured in two ways. Satisfaction with information about glaucoma will be measured using the Satisfaction with Information Scale [66] (four items). These questions were developed to assess how patients with hyperlipidemia felt about the type of information they received about their disease. The scale uses a seven point Likert scale to assess satisfaction with the amount of information received, the clarity of the information, how helpful the information was, and how well people now understood how to take their medications. The Satisfaction with Information scale will be given at the baseline study visit, and the 12-month study visit, at the conclusion of the eyeGuide intervention. Satisfaction with the eyeGuide intervention will also be measured at the 12-month study visit using the Client Satisfaction Questionnaire [67, 68] (three-item subscale, *α* = 0.91).

We will explore the effect of possible moderators and mediators of glaucoma medication adherence, such as total number of minutes of counseling, socio-demographic characteristics, visual function [69], health literacy [70, 71], severity of glaucoma, glaucoma symptoms [72], number of prescribed classes of glaucoma medications, medical co-morbidities, perceived stress [73], consideration of future consequences [74], barriers to glaucoma medication adherence [75], social support [76], depression [77, 78], glaucoma-related distress [79], hopefulness, confidence and motivation to manage glaucoma [32], and perceived benefit to glaucoma treatment [80]. These constructs will be measured using validated instruments or particular items from validated instruments to assess specific variables (Table 2). We will also explore the acceptability of and satisfaction with each level of the intervention through semi-structured interviews with participants. All participants will be interviewed with a semi-structured interview guide (Additional file 3). The number of minutes of counseling recorded alongside amount of booster training given will be used as an estimate of potential labor force costs to implementing the counseling program in the clinical setting.

**Table 2:** Scales for potential moderators and mediators of glaucoma medication adherence for exploratory analysis

Moderators	Mediators	Time point^a^	Original source
Visual function		1	National Eye Institute Visual Function Questionnaire (Mangione 2011)
Functional health literacy		1	Functional Health Literacy (Chew 2004)
Health literacy		2	Rapid Estimation of Adult Literacy in Medicine (Arozullah 2007)
Glaucoma symptoms		1	Glaucoma Symptom Scale (Lee 1998)
Perceived Stress		1	Perceived Stress Scale (Cohen 1983)
Consideration of future consequences		1	Consideration of Future Consequences Scale (Strathman 1984)
	Glaucoma-related distress	1, 3, 5, 6	Diabetes Distress Scale (Polonsky 2005)
	Barriers to glaucoma medication adherence	1, 3, 5, 6	Glaucoma Treatment Compliance Assessment Tool (Barker 2015)
	Social support	1, 5, 6	Diabetes Specific Social Support Needs (Rosland 2008)
	Depression	2, 5, 6	Patient Health Questionnaire-9 (Kroenke 2001)
	Hopefulness, confidence and motivation to manage glaucoma	1, 3, 5, 6	Hopefullness, Confidence and Motivation Rulers (Miller and Rollnick 2012)
	Perceived benefit to Glaucoma Treatment	1, 5, 6	Perceived Benefits of Treatment (Chao 2005)

^a^Time Point 1 = baseline (0 months), 2 = intervention 1 (alarms, 3 months), 3 = intervention 2 (eyeGuide, 6 months), 4 = eyeGuide follow-up visit (8 months), 5 = intervention end (12 months), 6 = end of adherence monitoring (24 months, end of study)

#### Study specific adverse event reporting

Any physical, social, economic, or psychological harm attributable to participation in this research study, if serious, will be reported to the IRB within 7 days and any non-serious adverse events will be reported with scheduled continuing review. Examples of possible adverse events include an injury occurring during a study visit, depression identified during a study visit, breach of confidentiality, or auto accident on the way to or from a study visit. Any unrelated deaths while in the study will be reported to the IRB with scheduled continuing review.

Any clinical depression identified during the study will be handled according to our depression screening protocol (Additional file 4). Participants who have issues with medication adherence may have problems because of underlying psychological distress. Therefore, we will use the patient health questionnaire (PHQ-9), a validated measure used clinically to assess for depression [77], during the 3-, 10-, and 12-month study visits so that all participants are screened for depression annually and if a participant is identified as having depressive symptoms they will be referred to appropriate care (see protocol, Additional file 4).

Any increases in IOP will be managed by the study principal investigator (PANC) and study team but are not considered reportable events to the IRB. When the participant’s IOP is checked, if it is above the goal pressure that has been set for them by their physician, the study team will contact their ophthalmologist through the electronic medical record and via email to let them know and determine an appropriate follow-up with their ophthalmologist. If the participant’s IOP is > 21 mmHg, the PI will be paged and the participant will be seen immediately in the glaucoma clinic by a physician for further management if medically indicated.

#### Data and safety monitoring plan

As the study has been deemed to have minimal risk by the Institutional Review Board (University of Michigan, Ann Arbor, MI), the Institutional Review Board has recommended that members of the study team will review study recruitment, adverse events, and compliance with the protocol every 6 months.

### Statistical design

#### Data analysis

The primary outcome, adherence, captured by the electronic monitors, will be a continuous measure from 0 to 100 representing the percent of glaucoma medication doses taken on time over a specific time period. Baseline adherence for the 3-month interval before any intervention will be investigated for the Hawthorne effect. Because simply monitoring a patient can cause them to temporarily change their behavior, even in the absence of actual intervention [81], we will plot adherence over time for each patient during the first 3 months of monitoring. Previous electronic medication monitoring for glaucoma medications demonstrated that the Hawthorne effect wore off after approximately 40 days of monitoring, which is why we chose to monitor participants for 90 days [82]. We will investigate plots for patterns of better adherence during the initial weeks of monitoring and worse adherence during the later weeks of baseline monitoring. Additionally, we will compare adherence in earlier vs. later weeks using paired *t* tests. If a significant Hawthorne effect is found, we will use the third month of monitoring data as a baseline adherence score. Although using the worst month of adherence as a baseline score could expose us to regression to the mean, we would expect outliers in adherence to be random throughout the 3-month period and not necessarily bias the results in only the last month of monitoring. Adherence at baseline and after the intervention will be compared with paired *t* tests for all pair-wise comparisons. Adherence 1 year after the end of all interventions will be evaluated for stability. This will be investigated with plots, and analyzed with paired tests and linear mixed regression models (LMM).

LMMs will be used to investigate predictors of adherence. Variables investigated will include participant characteristics and demographics, clinical measures of disease severity, and counseling and health literacy measures. Repeated measures logistic regression with generalized estimating equations (GEE) will also be used to investigate predictors of adherence >/=80%. The repeated measures analyses (LMM and GEE) will use adherence data gathered over all time points and account for the correlation within an individual over time. Moderators of glaucoma medication adherence will be investigated in the LMMs by interactions with time, to test the hypothesis that moderators will have different slopes of medication adherence over time [83]. Mediators of glaucoma medication adherence will be investigated with a series of LMMs [83]. First, the effect of the intervention on glaucoma medication adherence will be tested. Because all participants receive the intervention, this will be a test of slope of medication adherence over time (*Y*_medication adherence_ ~ *X*_time_). Next, the effect of the intervention on glaucoma medication adherence after adjustment for a mediator will be tested (*Y*_medication adherence_ ~ *X*_time_ + *X*_mediator)_. Lastly, the effect of the intervention on a mediator will be tested (*Y*_mediator_ ~ *X*_time_). After standardizing the effects from these three models, the direct, indirect, and total effects of the intervention will be estimated and Sobel’s test will be used to test the significance of the indirect effect.

We will adjust for weekly and seasonal trends when assessing other factors influencing adherence. We will use time series analysis using the 2 years of daily adherence data for each patient. Using Unobserved Component Models (UCMs), weekly and seasonal (yearly) cycles will be identified and tested in the context of a comprehensive regression model that adjusts for covariates such as participant characteristics and study intervention period. After adjustment, we will test for trends, e.g., a gradual improvement in adherence over time or a drop in adherence post-intervention [84, 85]. The SAS procedures TIMESERIES and UCM will be used for these analyses. The UCM procedure handles missing values in the dependent series and provides model diagnostics.

Secondary outcomes include comparing adherence measured by pharmacy refill data, self-report, and successful eye-drop instillation to electronic monitoring using Pearson’s correlations. Changes in glaucoma skills and energy for change will be assessed after each intervention component and at 1 and 2 years post-intervention and compared with paired *t* tests and McNemar’s tests for continuous and categorical measures. All analyses will be conducted with SAS software, version 9.4 (SAS Institute, Cary, NC, USA).

The semi-structured interviews will be recorded and transcribed verbatim. Two investigators will read the transcripts and identify major themes. The data will be interpreted qualitatively using grounded theory [86]. A code book will be generated defining codes for each theme. Two investigators will then code the transcripts. Any areas of disagreement will be discussed until consensus is reached or adjudicated by a third researcher [87]. The coded content will be tallied to weight the frequency of the themes. The transcripts will also be analyzed by participant group, those who did and those who did not improve from the intervention. Themes from these two groups will be compared in a joint display to try to identify reasons why the program was not successful among some participants to identify areas for programmatic improvement.

#### Sample size

We estimate that 46 participants will provide 80% power to detect at least a relative improvement of 15% in adherence (from an average pre- to post-intervention adherence rate of 0.7 to 0.8, and common standard deviation of 0.20) with a type-1 error rate of 5% and assuming a mild correlation of 0.3 between pre- and post-intervention adherences. We wanted adequate power to identify even a small effect size to evaluate whether this novel clinic-based intervention has any initial impact on adherence. This power calculation is based on the study by Okeke et al. in which patients < 75% adherent (measured with electronic monitors) were randomized to a standard counseling intervention vs. regular care [88]. This study demonstrated a 19% ± 20% improvement in adherence in the intervention group. We will aim to enroll 57 patients to allow for a 20% dropout rate; we anticipate this dropout rate given that we are targeting participants who are non-adherent to their recommended medication treatments. We aim to enroll 15 African-American patients to ensure that 25% of our patient population is African-American.

## Discussion

This project will develop and test technology-based, individually-tailored, behavior change programs designed to motivate people with glaucoma to improve their medication adherence. It uses a pre-post design and begins with automated reminders tailored to an individual’s preference and escalates to a counseling intervention aimed to help participants find their own solutions to adherence barriers and increase their motivation to take care of their glaucoma. The mixed methods design—quantitative adherence data and qualitative patient interview data—will enable us to test the intervention’s preliminary efficacy as well as identify areas for future improvement. The largest limitation to this pilot study is inherent in its design: it lacks a control arm. In addition, when participants know they are being monitored, they may change their behavior. We will investigate our data for this Hawthorne effect to estimate its impact on our results. These data will serve to alllow us to assess the feasibility of the intervention and test the plausibility of the intervention's impact prior to evaluating it in a randomized controlled clinical trial.

Creating a paradigm in which para-professional staff can increase the reach of physicians by providing personalized education and counseling that is individualized to the patient’s diagnosis, test results, treatment recommendations, and barriers to optimal adherence will help improve chronic disease self-management for glaucoma patients. Research in this area is necessary to create a robust evidence-based model for dissemination of improved best-practices for managing glaucoma. Strong evidence that this type of personalized behavioral intervention improves patient centered outcomes—such as satisfaction with health care provider communication and self-efficacy—alongside clinical outcomes—such as medication adherence and intraocular pressure fluctuation—will help inform policy decisions about reimbursing team-based care in the subspecialty setting in a parallel way to the reimbursement structure used with diabetic patients in primary care. Future work on integrating glaucoma education into the clinical encounter needs to be done to facilitate dissemination of any clinically useful results.

## Additional files

Additional file 1: Measures of self-reported adherence. (DOCX 14 kb)

Additional file 2: Modified One-Pass: tailored MI scoring for eyeGuide counselors. (DOCX 59 kb)

Additional file 3: Semi-structured exit interview guide. (DOCX 16 kb)

Additional file 4: Depression severity (PHQ-9 Score) and eyeGuide protocol. (DOCX 21 kb)

## Abbreviations

GEE: Generalized estimating equation
IOP: Intraocular pressure
LMM: Linear mixed model
MI: Motivational interviewing
UCMs: Unobserved component models
