# Supplementary material for: Personalized behavior change program for glaucoma patients with poor adherence: a pilot interventional cohort study with a pre-post design
Source: Pilot Feasibility Stud. 2018 Jul 23;4:128. doi: 10.1186/s40814-018-0320-6 (PMC6055343; doi:10.1186/s40814-018-0320-6)
Supplement: Supplementary file 1 — Measures of self-reported adherence. (DOCX 14 kb) [file 40814_2018_320_MOESM1_ESM.docx]

Additional file 1. Measures of Self-Reported Adherence

**Self-Reported Adherence Assessment:**

Chang Adherence Measure

1. “Over the past month, what percentage of your drops do you think you took correctly?”^a^

Morisky Medication Adherence Scale

1. Do you sometimes forget to take your glaucoma drops?

Yes (1) No (0)

2. Over the past two weeks, were there any days when you did not take your glaucoma drops?

Yes (1) No (0)

3. Have you ever cut back or stopped taking your glaucoma medication without telling your doctor because you felt worse when you took it?

Yes (1) No (0)

4. When you travel or leave home, do you sometimes forget to bring along your glaucoma medications?

Yes (1) No (0)

5. Did you take your glaucoma drops yesterday?

Yes (0) No (1)

6. When you feel like your eye pressure is under control, do you sometimes stop taking your eyedrops?

Yes (1) No (0)

7. Taking medication every day is a real inconvenience for some people. Do you ever feel hassled about sticking to your glaucoma treatment plan?

Yes (1) No (0)

8. How often do you have difficulty remembering to take all of your glaucoma medications?

All of the time (4) Usually (3) Sometime (2) Once in a while (1) Never/rarely (0)

^a^Reporting <95% adherence had a 2.6 times greater odds of having <80% adherence according to electronic medication monitoring in glaucoma patients.

Chang DS. Development and validation of a predictive model for nonadherence with once-daily glaucoma medications. Ophthalmology 2013; 120(7): 1396-1402.

^a^Morisky DE, Ang A, Krousel-Wood M, Ward H. Predictive Validity of a Medication Adherence Measure for Hypertension Control. Journal of Clinical Hypertension 2008; 10(5):348-354.

Krousel-Wood MA, Islam T, Webber LS, Re RS, Morisky DE, Muntner P. New Medication Adherence Scale Versus Pharmacy Fill Rates in Seniors with Hypertension. Am J Manag Care 2009;15(1):59-66.

Morisky DE, DiMatteo MR. Improving the measurement of self-reported medication nonadherence: Final response. J Clin Epidemi 2011; 64:258-263.
