# Supplementary material for: Personalized behavior change program for glaucoma patients with poor adherence: a pilot interventional cohort study with a pre-post design
Source: Pilot Feasibility Stud. 2018 Jul 23;4:128. doi: 10.1186/s40814-018-0320-6 (PMC6055343; doi:10.1186/s40814-018-0320-6)
Supplement: Supplementary file 2 — Modified One-Pass: tailored MI scoring for eyeGuide counselors. (DOCX 59 kb) [file 40814_2018_320_MOESM2_ESM.docx]

**Additional file 2 Modified OnePass: Tailored MI Scoring for eyeGuide Counselors**

## Introduction:

# This rating system was specifically designed for use with the eyeGuide as a way to measure both the eyeGuide counselors’ fidelity to Motivational Interviewing and their fidelity to the tailored patient education tool. The system is based off the OnePass fidelity tool developed by Fiona McMaster and Ken Resnicow. The OnePass is a validated user-friendly MI assessment and supervision tool, shown to have good inter-rater reliability, and is easily adaptable to different clinical contexts and use of MI (McMaster, F., Resniow, K., (2015). Validation of the one pass measure for motivational interviewing competence. Patient Education and Counseling. Vol 98, 4, pp499-505.)

## Scoring Guidelines:

Scoring guidelines are provided below. The tool is composed of two parts, one to evaluate the counselor’s fidelity to Motivational Interviewing using a series of likert scale questions, and the other part is a series of binary yes/no questions to evaluate the counselors fidelity to the tailored patient education tool. Although many of these tasks are MI in nature, they are scored on a binary instead of Likert scale because the tasks are written into the eyeGuide structure. The eyeGuide is structured to guide counselors on how to appropriately use some of the MI skills such as the rulers and to remind counselors to reflect back what the patient said before moving on to quickly. It is expected that the counselor will score yes on all 18 tasks, but a score ≥75% is acceptable. When computing the score items scored as “NA” should be excluded.

To score the counselors fidelity to MI we encourage raters to use the entire scoring range on the Likert scale of 1 through 7. A “Not Applicable” option has also been provided for instances when despite the best intentions of the counselor the participants had minimal to no response. A score of 4 is considered average counselor competence. To assign a score of 1, it is not necessary that the counselor be abusive or incompetent, but rather only that s/he exhibited the low end of acceptable response. Similarly, a score of 7 would not require that the counselor be an expert in MI like MI creator Bill Miller, but rather should be at the high end of competent. A column for “comments/high points” is provided for coders to indicate key counselor statements that were used to generate their score for that item, and to assist in giving feedback to the practitioner. This column may also be used to jot down notes or keep a “running tally” of counselor behavior to allow the rater to compute a final score for that item. When computing overall means, exclude items scored as “NA”.

For scoring the global performance metric of patient versus counselor talk time, the amount of talk time should be scored as the conversation time during the prompts sections of the eyeGuide and not during the required written tailored education portions that the counselor has to read aloud to the patient.

**Passing score for counselor’s fidelity to tailored patient education tool of the eyeGuide:**

**Score ≥75% (18 questions)**

**Passing score for counselor competence in Motivational Interviewing Skills:**

**Mean of 5 (17 total questions)**

MI trainer: eyeGuide Counselor: Patient: Date recorded: Date Scored:

**Scoring for eyeGuide Counselors: Modified OnePass**

| **How effectively did the Counselor…** | **Comments/High Points** | **Poor/ Never** | | | **Good/Often** | | | **Excellent/ Always** | | |
| --- | --- | --- | --- | --- | --- | --- | --- | --- | --- | --- |
| ***GENERAL MOTIVATIONAL INTERVIEWING SKILLS*** | | | | | | | | | | |
| Notes for patient experience, change talk and sustain talk: | | | | | | | | | | |
|  |  | | **1** | **2** | **3** | **4** | **5** | **6** | **7** | N/A |
| 1. …use open-ended and benign closed questions? |  | |  |  |  |  |  |  |  |  |
| 1. …use reflective listening? (providing simple and complex reflections) |  | |  |  |  |  |  |  |  |  |
| 1. …express empathy/warmth/acceptance? |  | |  |  |  |  |  |  |  |  |
| 1. …affirm & build efficacy by affirming effort, commitment, and attempts? |  | |  |  |  |  |  |  |  |  |
| 1. …respond appropriately to glaucoma patient’s affect and stated emotions? |  | |  |  |  |  |  |  |  |  |
| 1. ..avoid any judgmental statements about anything the glaucoma patient says? |  | |  |  |  |  |  |  |  |  |
| 1. …avoid providing unsolicited advice and/or information? |  | |  |  |  |  |  |  |  |  |
| 1. …support patient autonomy (volition) |  | |  |  |  |  |  |  |  |  |
| 1. …provide summaries of each section? |  | |  |  |  |  |  |  |  |  |
| 1. …help the glaucoma patient identify barriers to drop adherence throughout? |  | |  |  |  |  |  |  |  |  |
| 1. …elicit the patient’s motivations, values and strengths throughout? |  | |  |  |  |  |  |  |  |  |
| ***SUMMARY*** | | | | | | | | | | |
| 1. …demonstrate MI spirit throughout? |  | |  |  |  |  |  |  |  |  |
| 1. …overall, how well did the eyeGuide counselor use MI in this session? |  | |  |  |  |  |  |  |  |  |
| ***GLOBAL PERFORMANCE METRICS*** | | | | | | | | | | |
| 1. Glaucoma patient vs. Counselor Talk Time | GOAL >50% | | < 50% | | | ~50% | | | >50% | |
| 1. Reflection to Question ratio | GOAL > 2:1 | | <1:1 | | | ~1:1 | | | >2:1 | |
| 1. Percent Open Questions (of total Q) | GOAL >70% | | <50% | | | 50-70% | | | >70% | |
| 1. Percent Complex Reflections (of total reflections) | GOAL > 50% | | <25% | | | 25-50% | | | >50% | |

| ***TAILORED PATIENT EDUCATION TOOL: COMPLETED EYEGUIDE TASKS*** | | | | |  |  |
| --- | --- | --- | --- | --- | --- | --- |
|  | **Comments/High Points** | **Yes No** | | | **N/A** |  |
| ***LEARN SECTION*** | | | | | | |
| 1. If a phone session – made sure the patient was free to talk and, if not, scheduled another time/day to call? |  | |  |  |  |  |
| 1. Open with warm greeting and self-introduction? |  | |  |  |  |  |
| 1. Give glaucoma patient time to describe their experience? |  | |  |  |  |  |
| 1. Input client specific pictures and test results into eyeGuide? |  | |  |  |  |  |
| 1. Provide a summary of the learn section? |  | |  |  |  |  |
| ***USE SECTION*** | | | | | | |
| 1. Watched patient put in drops? |  | |  |  |  |  |
| 1. Reviewed new methods to put in drops? |  | |  |  |  |  |
| 1. Watched patient demonstrate use of new methods? |  | |  |  |  |  |
| 1. Summarize their plan for improving eye drop instillation? |  | |  |  |  |  |
| ***GOALS SECTION*** | | | | | | |
| 1. Elicit importance using ruler? |  | |  |  |  |  |
| 1. Elicit confidence using ruler? |  | |  |  |  |  |
| 1. Elicit motivations and values and connect these to drop adherence? |  | |  |  |  |  |
| 1. Elicit patient strengths and help them identify their personal qualities linked to these strengths that they can use for better drop adherence? |  | |  |  |  |  |
| 1. Help glaucoma patient identify barriers to drop adherence? |  | |  |  |  |  |
| 1. Elicit problem-solving with the use of the videos? |  | |  |  |  |  |
| 1. Provide a summary of the goals section? |  | |  |  |  |  |
| ***PLAN SECTION*** | | | | | | |
| 1. Summarize the entire session and the patients plan? ***SECTION*** |  | |  |  |  |  |
| 1. Help patient to identify specific action steps to help them get started in the next few days? |  | |  |  |  |  |

| ***TOTAL SCORES*** | | | | |
| --- | --- | --- | --- | --- |
| LIKERT SCALE QUESTIONS 1-13 | Goal mean of 5 | <5 | >5 | 7 |
| GLOBAL METRICS 14-17 | See scoring chart above | | | |
| BINARY QUESTIONS 18- 35 | Goal 18 yes responses (if N/A score as yes) | <11 | >15 | 18 |

Total recording time:

By Section

- Learn section:
- Use section:
- Goals section:
- Plan section:

DETAILED SCORING GUIDE

| **ITEM** | | | **1** | | | **4** | | **7** | | | | NA/Details | |  |
| --- | --- | --- | --- | --- | --- | --- | --- | --- | --- | --- | --- | --- | --- | --- |
|  | |  |  | | | | | | | | | | |  |
| **1** | | UE OPEN ENDED CLOSED QUESTIONS | Frequent, “nasty” judgmental Closed vs. Open-Ended Questions. (*e.g., Do you miss a lot of doses? Have you always forgotten your eye drops?)*  Frequent use of questions when reflection possible.  May be occasional open questions | | | Some closed questions  Some use of questions when reflection possible.  Any closed questions are benign | | Questions consistently open (*e.g. Tell me about your experience with glaucoma? What if any problems are you have having with physically putting in your drops?)*  Rare use of questions when reflection possible | | | |  | |  |
|  | | | | | | | | | | | | | |  |
| **2** | REFLECTIVE LISTENING | | Mostly simple reflections (surface/content reflections)  Repeats what client said, adds minimal meaning to client statements  Parrots, Restates  Frequently uses questions instead of reflections  Does not provide space for the client to share their experience during the session  Unskilled or inappropriate attempts made to redirect tangential or verbose clients. Or no redirection is made at all | Some complex (meaning/feeling) reflections  Reflections add somewhat to client’s statements  Some rephrases/paraphrases  Occasionally uses questions instead of reflections  Provides some space for the client to share their experience during the session  Less skilled or appropriate attempts made to redirect tangential or verbose clients | | | Consistent complex feeling reflections/rolling with resistance  Reflections significantly add to client statements  Consistent rephrases/paraphrases/reframing  Relies almost exclusively on reflections  Provides significant space for the client to share their experience during the session  Appropriately and skillfully redirects tangential or verbose clients | | | |  | | |  |
|  | | | | | | | | | | | | | |  |
| ITEM 1 4 7 NA/Details | | | | | | | | | | | | | |  |
| **3** | EXPRESS EMAPTY/ WARMTH/ACCEPTANCE | | Did not express empathy, warmth or acceptance towards the client | Was warm, empathetic, and accepting towards the client for part of the session | | | Expressed empathy, warmth and acceptance towards the client during the entirety of the session | | | |  | | |  |
|  | | | | | | | | | | | | | |  |
| **4** | AFFIRM CLIENT EFFORTS | | May affirm client’s position occasionally, but in the form of praise rather than ‘evidence-based’ affirmations. *e.g. ‘Good job’ ‘you’re a great glaucoma patient’* | Some ‘evidence-based’ affirmations as well as some praise. These may include phrases such as *‘that shows me you care about your health’*  Reinforce effort, attempts, and commitment attempts when there has not been full success | | | Appropriate affirmations throughout  Commenting thoughtfully on the patients effort, attempts, commitment, strengths, and motivations | | | |  | | |  |
|  | | | | | | | | | | | | | |  |
| **5** | RESPOND APPROPRIATELY TO CLIENT AFFECT | | Glosses over or ignores client affect | Partially acknowledges and/or reflects client affect  Does not use intense feeling words | | | Fully acknowledges and reflects client affect  Uses intense feeling words | | | | ***NA: Client does not express adequate verbal/non-verbal emotional content to adequately assess*** | | |  |
|  | | | | | | | | | | | | | |  |
| **6** | AVOID JUDGEMENTS | | “Wrestles” With the individual; feels like there is a constant struggle between client and counselor  Argues back against client  Makes judgmental statements about the patients experiences, knowledge or efforts | Ignores clients frustrations and reasons against change ***AND/OR*** reasons for not changing  Does not counterpunch  Does not make judgmental comments about the patients experiences, knowledge or efforts | | | Allows client to be ambivalent ***AND*** acknowledges reasons against change ***AND/OR*** reasons for not changing  Does not counterpunch  Does not make judgmental comments about the patients experiences, knowledge or efforts | | | |  | | |  |
|  | | | | | | | | | | | | | |  |
| **7** | AVOID PROVIDING UNSOLICITED ADVICE AND/OR INFORMATION | | Frequently gives unsolicited advice/info  Does not seek client understanding  Does not obtain permission to give information or advice  Misses opportunity to use Elicit-Provide-Elicit  Responds to suggestions with *‘Yes, but…’*  Tells the client what to do; gives advice | Occasionally gives unsolicited advice/info  Partially seeks clients understanding  Sometimes asks for permission to give advice or information. Uses Elicit-Provide-Elicit  Gives occasional advice | | | Does not give unsolicited advice/info  Thoroughly seeks client understanding.  Offers information or advice only with permission. Uses Elicit-Provide-Elicit  Gives little or no unsolicited advice  Undersells advice | | | | ***When getting permission, overuse of ‘is this ok?’ type phrases will obtain a lower score than counselors who use a variety of linguistic forms.*** | | |  |
|  | | | | | | | | | | | | | |  |
| **8** | SUPPORT CLIENT AUTONOMY | | Authoritative style (style)    Does not emphasizes client autonomy and volition (style)  Provides no choice (strategy) | Mix of authoritative and egalitarian  Partially Emphasizes client autonomy and volition  Provides some choice | | | Strongly egalitarian  Fully Emphasizes client autonomy and volition  Provides frequent choices | | | |  | | |  |
|  | | | | | | | | | | | | | |  |
|  | **ITEM** | | **1** | **4** | | | **7** | | | | NA/Details | | |  |
| **9** | PROVIDE SUMMARIES OF EACH SECTION | | Does Not Summarize main session content  Does Not Ask client to edit summary  Does Not provide balanced summary of pros and cons | Partially summarizes main session content or does not include patient values, strengths, importance  Seeks some feedback about summary  Provides unbalanced summary of pros and cons or puts sustain talk last | | | Fully summarizes main session content as well as patient’s values, and strengths and what is important to them  Seeks feedback about summary  Provides balanced summary of pros and cons  Summarizes sustain talk first and ends with change talk | | | |  | | |  |
|  | | | | | | | | | | | | | |  |
| **10** | IDENTIFY BARRIERS | | Did not elicit client barriers to eye drop use  Counselor identified barriers and chose barrier videos to watch without collaboration from the client | Elicited from the client at least one barrier  Counselor elicited from patient the barriers videos to watch | | | Elicited from the client all of the barriers they are experiencing related to eye drop use  Counselor elicited from patient the barriers videos to watch | | | |  | | |  |
| **ITEM** | | | **1** | **4** | | | **7** | | | | NA | | |  |
| **11** | EFFECTIVELY ELICITS AND ADDRESS VALUES AND MOTIVATIONS THROUGHOUT | | Did not fully elicit values, motivations or strengths  Does not probe or reflect client values and strengths  Does not properly probe for connection of patient values/goals/strengths to eye drop behavior  Does not encourage client to link values/goals to eye drop behavior | Partially elicited and/or discussed values/goals/strengths  Moderately skilled probe for connection of values/goals/strengths and eye drop behavior    Helps client link values/goals/strengths to eye drop behavior ***BUT*** does not skillfully elicit additional change talk | | | Fully elicited and/or discussed values/goals/strengths  Highly skilled probe for connection of values/goals/strengths and eye drop behavior    Helps client link values/goals/strengths to eye drop behavior ***AND*** skillfully elicits additional change talk | | | |  | | |  |
|  | | | | | | | | | | | | | |  |
| **12** | DEMONSTRATE MI SPIRIT | | Does not collaborate, show empathy, support autonomy, acceptance, and client-centeredness during the session | | Partially collaborates, shows empathy, supports autonomy, acceptance, and client-centeredness during the session | | | | Fully collaborates, shows empathy, supports autonomy, acceptance and client-centeredness during the session | | | | ***NB: This is a composite of several other measures, but should be assessed on overall impression*** | |
|  | | | | | | | | | | | | | | |
| **13** | HOW WELL DID THE COUNSELOR CONDUCT THE SESSION? | | Poor MI Skills | | Moderate MI Skills | | | | High MI Skills | | | | ***NB: This is a composite of several other measures, but should be assessed on overall impression*** | |
| ITEMS 14-17 | | | | | | | | | | | | | | |
| SEE GLOBAL METRICS SCORING CHART | | | | | | | | | | *These are subjective estimates of MITI performance criteria* | | | |  |
|  | | | | | | | | | | | | | | |
