# Supplementary material for: Personalized behavior change program for glaucoma patients with poor adherence: a pilot interventional cohort study with a pre-post design
Source: Pilot Feasibility Stud. 2018 Jul 23;4:128. doi: 10.1186/s40814-018-0320-6 (PMC6055343; doi:10.1186/s40814-018-0320-6)
Supplement: Supplementary file 3 — Semi-structured exit interview guide. (DOCX 16 kb) [file 40814_2018_320_MOESM3_ESM.docx]

Additional file 3. Semi-Structured Exit Interview Guide

Introduction: You are participating in a 2-year study about the eyeGuide education program. You took many surveys, kept your glaucoma medication in special bottles, had in-person sessions with the glaucoma counselor and had phone check-ins with the glaucoma counselor. We want to hear your opinion about your eyeGuide sessions with the glaucoma counselor, both in-person and over the phone.

1. What were your experiences participating in this program?
2. What did you think of the website (show the website)?

Which were your favorite parts?

2. Was the glaucoma counselor helpful? Easy to talk to?

3. Did you ever go back and login to the website on your own?

4. Where did you post your plan page?

Was it helpful?

5. What did you think about the phone calls?

6. What was your experience of hearing your adherence number?

B. What didn’t you like about the program?

1. What were some barriers to participating in the program?

2. What were some barriers to participating in the phone calls?

3. What was the most difficult part about participating?

C. How, if at all, did you eye drop use change while you were going through the eyeGuide program?

1. What element of the program was most helpful in making this change?

The education session, being able to ask Laurie questions, writing questions for your doctor, knowing your adherence level, having alerts on your bottle, having text message or voice message reminders when you forgot a dose?

D. If we could do the return visits or even the first visit over the phone so that you wouldn’t have to travel to clinic, what would you think?

1. Would you be more likely to participate?

2. Do you think it would be a worse experience if you weren’t interacting with Laurie in-person?

E. Do you have any feedback for us on how we could improve the program in the future?
