# Supplementary material for: Personalized behavior change program for glaucoma patients with poor adherence: a pilot interventional cohort study with a pre-post design
Source: Pilot Feasibility Stud. 2018 Jul 23;4:128. doi: 10.1186/s40814-018-0320-6 (PMC6055343; doi:10.1186/s40814-018-0320-6)
Supplement: Supplementary file 4 — Depression severity (PHQ-9 Score) and eyeGuide protocol. (DOCX 21 kb) [file 40814_2018_320_MOESM4_ESM.docx]

Additional file 4. Depression Severity (PHQ-9 Score) and eyeGuide Protocol

Depression Protocol

1. Patients scheduled to take the PHQ-9 measure at the 3, 12 and 24 month in-person visits
2. Tally up total scores and follow suggested protocol

Table 4. PHQ-9 Scores and Proposed Treatment Actions *

| *PHQ-9 Scores* | *Depression Severity* | *Proposed Treatment Actions* | ***EyeGuide Protocol*** |
| --- | --- | --- | --- |
| 0 – 4 | None-minimal | None | **Do nothing, tell patient they have minimal to no depression based on this measure**  **“Based on the questionnaire you completed, it appears that you are not struggling with any depression symptoms at this time”** |
| 5 – 9 | Mild | Watchful waiting; repeat PHQ-9 at follow-up | **Do nothing, tell patient they may have mild depression based on this measure**  **“Based on the questionnaire you completed, it appears that you may be struggling with some mild depression symptoms at this time”** |
| 10 – 14 | Moderate | Treatment plan, considering counseling, follow-up and/or  pharmacotherapy | **Ask the patient if they are interested in talking with a social worker on staff or would like a list of mental health/counseling resources**  **“Based on the questionnaire you completed, it appears that you are struggling with some depression symptoms, would you be interested in talking with our social worker on staff about your mood? If it’s ok, I would also like to provide you with a list of counseling resources that you can take with you.”** |
| 15 – 19 | Moderately Severe | Active treatment with pharmacotherapy and/or psychotherapy | **Recommend that the patient talk with the social worker on staff and ask if they would like a list of mental health/counseling resources**  **“Based on the questionnaire you completed, it appears that you are struggling with some significant depression symptoms. I you are willing; I would recommend you talk with our social worker on staff about how you have been feeling, how does that sound to you? If it’s ok, I would also like to provide you with a list of counseling resources that you can take with you.”** |
| 20 – 27 | Severe | Immediate initiation of pharmacotherapy and, if severe impairment or poor response to therapy, expedited referral to a mental health specialist for psychotherapy and/or collaborative management | **Recommend that the patient talk with the social worker on staff and ask if they would like a list of mental health/counseling resources**  **“Based on the questionnaire you completed, it appears that you are struggling with some significant depression symptoms. I you are willing; I would recommend you talk with our social worker on staff about how you have been feeling, how does that sound to you? If it’s ok, I would also like to provide you with a list of counseling resources that you can take with you.”** |
| Question 9: Suicidal Ideation | Moderate - Severe | If the patient answers “several days”, “more than half the days”, or “nearly every day”. Active treatment with pharmacotherapy and/or psychotherapy | **Page social worker on staff to come and speak with the patient prior to them leaving the session with you. The social worker will further screen the patient for suicidal ideation/plan/intent and offer counseling referrals, or if necessary transfer to the U of M Psychiatric Emergency Services (PES)**  **“Based on the questionnaire you completed, it appears that you are struggling with some significant depression symptoms, and are having thoughts of suicide or harming yourself. For this research study it is protocol that we page our social worker on staff to come and speak with you to make sure that you are able to keep yourself safe. I will page them now, and I would also like to give you this list of counseling resources and emergency numbers to take with you.”** |
| Question 10:  Difficulty level | Moderate- Severe | If the person answers very difficult or extremely difficult | **Recommend that the patient talk with the social worker on staff and ask if they would like a list of mental health/counseling resources**  **“Based on the questionnaire you completed, it appears that you are struggling with some significant depression symptoms. I you are willing; I would recommend you talk with our social worker on staff about how you have been feeling, how does that sound to you? If it’s ok, I would also like to provide you with a list of counseling resources that you can take with you.”** |
| Question 11:  Mental Health Care | Mental Health Care | If the person answers yes to both question 11 and 11A | **No nothing, reflect that they are actively working to treat their mental health condition**  **“Based on your answers in the questionnaire it appears that you are currently working to treat your mental health, and feel that you are receiving good care.”** |
| Question 11A:  Satisfaction with Mental Health Care | Satisfaction with Mental Health Care | If the person answers no to question 11A | **Ask if they would like a list of other mental health/counseling resources**  **“Based on the questionnaire you completed, it appears that you are receiving mental health care, but that it may not be fully meeting your needs, if it’s ok with you I would like to provide you with a list of other counseling resources that you can take with you.”** |

* From Kroenke K, Spitzer RL, Psychiatric Annals 2002;32:509-521
https://phqscreeners.pfizer.edrupalgardens.com/sites/g/files/g10016261/f/201412/instructions.pdf

- If the patient responds yes – that they would like to speak with a social worker, contact the social worker on staff to speak with them in the moment, or, if not urgent, request that the social worker call the patient to set up a time to speak with them
- Offer the patient a printed list of mental health resources (see below)

**For the social worker on staff:**

If paged to speak with an eyeGuide study patient. Review with the patient their PHQ-9 scores (eyeGuide counselor will give you a hard copy of the patients PHQ-9). Complete a full suicide assessment (ideation/plan/intent) and offer counseling referrals, or if necessary transfer the patient to the U of M Psychiatric Emergency Services (PES)

Document the visit, including the patients PHQ-9 score, the plan moving forward, and document the risk assessment as follows (selecting the appropriate items in each factor area):

RISK ASSESSMENT:

- Static Risk Factors: (none, previous suicide attempts, previous aborted suicide attempts, history of self-injurious behavior, family history of suicide, recent discharge from inpatient or PES, white male over age 60, male age 15-19, history of mood, psychotic, or substance use disorder, recent change in provider, history of bullying or being bullied, history of childhood abuse, peers with recent suicide attempts)

- Modifiable Risk Factors: (none, current mood, psychotic, or substance use disorder, current suicidal ideation, global insomnia, recent increase in substance use, disruptive disorder, purposelessness, intense anxiety, panic attacks, feeling trapped, hopelessness, social isolation, impulsivity, mood swings, recent humiliation or shame, ongoing medical illness, loss of significant relationship, limited coping skills, strained parent-child relationship, legal problems, difficulty in school)

- Protective Factors: (none, strong coping skills, religious prohibition against suicide, high frustration tolerance, African-American female, responsibility to loved ones, positive therapeutic relationship, social support, high family cohesion, future oriented, hopeful, convincing commitment to safety, fear of pain, disability, or death)

- Imminent Risk Factors: (none, access to firearms, suicide plan, intention to carry out plan, belief in lethality of plan)

- Overall Risk: (low, moderate, high)

- Plan: (Referral to mental health services, ongoing care with a current mental health provider, transfer to PES)

**Documentation:**

Following the visit, the eyeGuide counselor should document the patients PHQ-9 score and the plan in their chart as follows (filling in the italicized sections):

*Patient name*, completed the PHQ-9 (Patient Health Questionnaire on depression severity) during today’s session and scored a *#* indicating that the patient has *state severity* depression symptoms*.* The counselor shared with the patient that their survey indicates that they are struggling with *state level* depression symptoms, and it was recommended that the *patient talk with a social worker on staff about their mood*. *The patient was also given a list of contact information for mental/health and counseling resources in the area.*

(If appropriate) Social worker, *write name*, was contacted and met with the patient to review their PHQ-9 scores, and further evaluate the patients suicidal risk.

**Mental Health Resources**

University of Michigan Department of Outpatient Psychiatry

Rachel Upjohn Building, East Medical Campus

4250 Plymouth Road

Ann Arbor, MI 48109

(734) 764-0231

Website: http://www.psych.med.umich.edu/patient-care/ambulatory-psychiatry/

The University Psychological Clinic

500 E. Washington St., Suite 100

Ann Arbor, MI 48104

(734) 764-3471

Website: http://mari.umich.edu/psych-clinic/

Huron Valley Consultation

2750 South State Street

Ann Arbor, MI 48104

(734) 662-6300

Website: <http://www.huronvalleyconsult.org/>

Catholic Social Services

4925 Packard

Ann Arbor, MI 48108-1521

(734) 926 -0155.

Website: http://csswashtenaw.org/counseling/bhs/

Psychology Today

Find detailed professional listings for Psychologists, Psychiatrists, Therapists, Counselors, Support Groups and Treatment Centers in the United States and Canada.

<https://therapists.psychologytoday.com/rms>

**If you are having a Psychiatric Emergency**

Call 911

Or go to the University of Michigan Department of Psychiatric Emergency Services (PES)

- PES provides emergency/urgent walk-in evaluations and crisis phone services 24 hours a day, 7 days a week, for adults. Phone number: (734) 936-5900.
- Location: University Hospital, 1500 East Medical Center Drive, Reception: Emergency Medicine, Ann Arbor, MI 48109
- <http://www.psych.med.umich.edu/patient-care/psychiatric-emergency-service/>

Call the: National Suicide Prevention Lifeline: 1-800-273-TALK (8255)
